# An Alternative Method for Treating Dumping Syndrome Using Hemoclips

**DOI:** 10.7759/cureus.14869

**Published:** 2021-05-06

**Authors:** Arda Yavuz, Kübra Akan, Celal Ulaşoğlu, İlyas Tuncer, Yaşar Çolak

**Affiliations:** 1 Gastroenterology and Hepatology, Istanbul Medeniyet University Göztepe Research and Training Hospital, Istanbul, TUR; 2 Gastroenterology, Istanbul Medeniyet University Göztepe Research and Training Hospital, Istanbul, TUR

**Keywords:** dumping syndrome, obesity, hemoclips, argon plasma coagulation, gastroscopy

## Abstract

Surgeries for obesity can lead to complications. Dumping syndrome is one such complication caused by the quick passage of hyperosmolar chyme from the stomach to the duodenum. Mild cases can be cured with dietary modification and medical treatment. However, refractory cases may need invasive treatment options, such as transoral outlet reduction or surgery. We successfully treated a 48-year-old female with dumping syndrome, using a combination of argon plasma coagulation and hemoclips to narrow the pyloric lumen. We suggest that this new technique could be a cheap and easily accessible alternative to surgery, especially in countries where the specialised devices needed to treat such cases are unavailable.

## Introduction

Obesity is a common disease affecting humans in the modern world. An increasing number of gastric bypass or gastric sleeve surgeries is being performed due to the high prevalence of obesity. Both these surgeries may lead to complications such as vitamin or iron deficiency, stricture, increased gallstone prevalence, protein and calorie malnutrition, leakage, perforation of the stomach or intestines, internal bleeding, profuse bleeding of the surgical wound, hernia, and dumping syndrome (DS).

DS is a known complication of gastric bypass or sleeve gastrectomy. The incidence of DS has been increasing and has been reported to be as high as 40% in some cases. DS can be classified into two types depending on the timing of symptoms: early DS (symptoms occur within an hour after a meal) and late DS (symptoms occur up to three hours after a meal). Both types are characterized by gastrointestinal and vasomotor symptoms, including tachycardia, fatigue, syncope, and, sometimes, shock and seizures. Due to quick hyperosmolar passage of chyme from the stomach to the duodenum, neurohumoral mechanisms are activated in both types of DS. The Sigstad diagnostic score system is based on symptoms and is used to identify DS versus non-DS. A score greater than 7 suggests DS [1].

Diet modification is the first recommendation for treating DS. Guar gum or pectin can delay gastric emptying, while acarbose and somatostatin analogues are beneficial in mild cases. There is limited data on the use of diazoxide, nifedipine, and exendin 9-39 as a medical treatment for DS [2]. For refractory cases, transoral outlet reduction (TORe) can be an alternative to surgery. In this case report, when presented with a patient with DS, we performed a method like TORe; however, instead of gastric suturing, we used hemoclips as a practical alternative.

## Case presentation

A 48-year-old Turkish female underwent sleeve gastrectomy for obesity in 2014. Before the surgery, she was 127 kg, and she lost weight following the surgery. When she was 62 kg, she started to suffer from nausea, meteorism, borborygmi, dyspnea, flutter, hypoglycemia, and dizziness in the first hour following a meal. After that, she gained weight and was 78 kg due to hypoglycaemic episodes.

**Figure 1:**
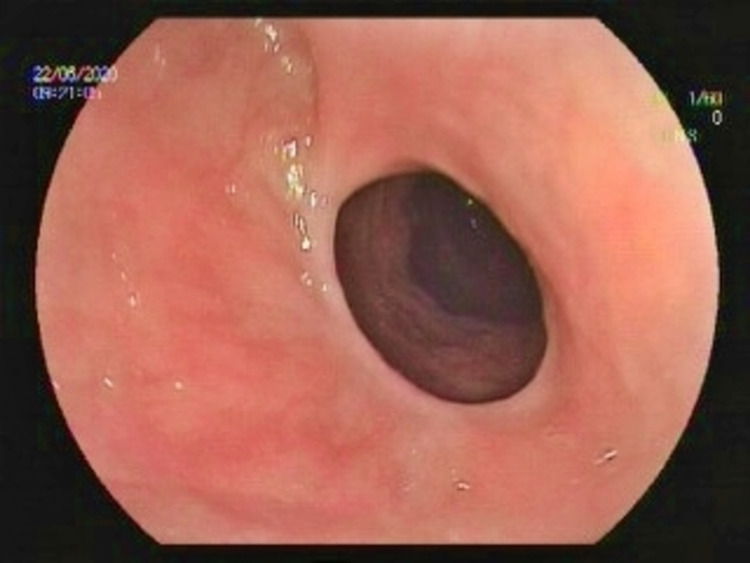
Deformed pylorus

**Figure 2:**
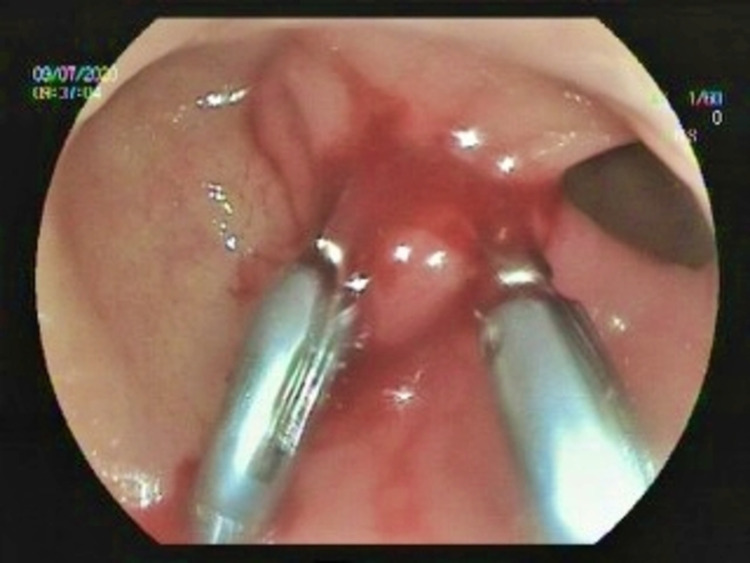
Use of endoscopic hemoclips

She was admitted to our facility in 2020 because of her persistent worsening symptoms. Her laboratory results and vital signs were normal. She did not take any medication for DS. On gastroscopy, we observed that the pylorus was deformed and widely open (Figure 1). Moreover, her Sigstad score was 17. We used argon plasma coagulation (APC) to repair the anterior and apical surface of the pylorus. Then, with two hemoclips, we narrowed the pyloric canal to retain an opening of 10 mm in diameter (Figure 2). After the procedure, the patient’s all symptoms were immediately resolved.

**Figure 3:**
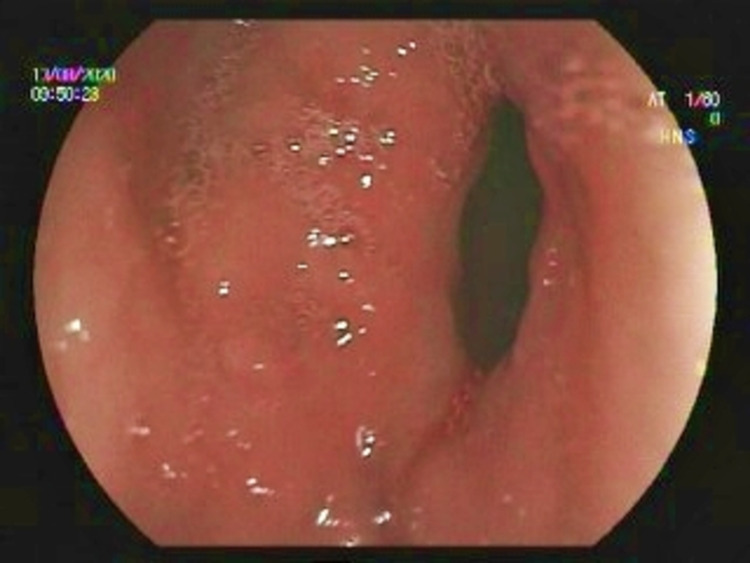
Narrow pyloric canal five weeks after the treatment

After five weeks, we performed a control gastroscopy. We found that the mucosa had healed, the clips were gone, pyloric opening was narrower, and the Sigstad score was 4. Six months following the procedure, our patient displayed no symptoms. Thus, we had successfully treated DS in our patient by using hemoclips (Figure 3).

## Discussion

Our patient developed DS after gastric surgery. We successfully treated her by using hemoclips as an alternative method to TORe. Her symptoms resolved after the treatment, and the Sigstad score reduced from 17 to 4. We checked the gastric outlet region during control gastroscopy five weeks later.

There are few endoscopic treatment alternatives to surgery for patients who are refractory to medical treatment for DS. One of them is APC, which is a non-contact electrocoagulation method that uses ionized gas. It is beneficial, with limited chances of tissue penetration in critical areas such as the colon and duodenum. APC also has a high proliferative effect on the circumferential area. With repeated sessions, this technique helps to reduce the diameter of the anastomosis. It also reduces the chances of weight regain [3].

Other methods for treating DS include endoluminal injection of sodium morrhuate into the anastomosis [4-5], use of the Bard Endocinch device [6], use of StomaphyXTM (EndoGastric Solutions, Inc., Redmond, WA, USA) [7], the ROSE procedure [8], the OverstitchTM (Apollo Endosurgery, Inc., Austin, TX, USA) [9], and the OTSC clip [10].

TORe is another method that can be conveniently used in such cases. The suturing device in this method revises the gastrojejunal anastomosis. In a German study, 115 patients had undergone TORe, and all cases were technically successful [11]. All these improved options have reduced the need for surgery in such cases.

In our case, we first used APC on one side of the pylorus for inducing mucosal healing. Then, with endoscopic hemoclips, we narrowed the pylorus. A combination of endoscopic hemoclips with APC is a practical alternative to all the methods mentioned above because the devices required for most methods are not available in many countries. Moreover, the method we have described here is more beneficial as there are no requirements for any alternative devices or additional sessions. We have reported this case as we want to highlight this cost-effective and easily accessible technique, which can eliminate the need for surgery in cases of DS after gastric surgeries for obesity.

## Conclusions

There are few endoscopic treatment alternatives, especially for the patient who has deformed pylorus and has the dumping syndrome. In that cases, argon plasma coagulation and hemoclips combination can be a successful and cost-effective alternative.
